# Effect of individualized end-inspiratory pause guided by driving pressure on respiratory mechanics during prone spinal surgery: a randomized controlled trial

**DOI:** 10.3389/fmed.2025.1537788

**Published:** 2025-04-09

**Authors:** Ting Zhang, Feng Lv, Shuangyu He, Yuntian Zhang, Li Ren, Juying Jin

**Affiliations:** Department of Anesthesiology, The First Affiliated Hospital of Chongqing Medical University, Chongqing, China

**Keywords:** end-inspiratory pause, driving pressure, lung protective ventilation strategy, pulmonary compliance, prone spinal surgery

## Abstract

**Purpose:**

The prone position is commonly used in spinal surgery, but it can lead to decreased lung compliance and increased airway pressure. This study aimed to evaluate the effect of individualized end-inspiratory pause guided by driving pressure on respiratory mechanics in patients undergoing prone spinal surgery.

**Methods:**

A randomized controlled trial was conducted from August to October 2023. Patients scheduled for elective prone spinal surgery were randomly assigned to either a study group, receiving individualized end-inspiratory pause, or a control group, receiving a fixed end-inspiratory pause (10% of total inspiratory time). Mechanical ventilation parameters, including tidal volume, plateau pressure, driving pressure, and peak pressure, were recorded at different time points. Arterial blood gases were collected at baseline and at specified intervals.

**Results:**

Data from 36 subjects (18 in each group) were included in the final analysis. The study group exhibited a significant increase in respiratory system compliance (*P* < 0.05) and improved intraoperative oxygenation (*P* < 0.05). In addition, the individualized end-inspiratory pause significantly decreased plateau pressure (*P* < 0.05) and driving pressure (*P* < 0.05) compared to the control group.

**Conclusion:**

The individualized end-inspiratory pause guided by driving pressure effectively optimized pulmonary compliance and improved oxygenation during prone spinal surgery. These findings suggest that this ventilation strategy may enhance respiratory mechanics and reduce the risk of postoperative pulmonary complications.

## Introduction

The prone position is frequently used in spinal surgery due to its advantages in surgical exposure. However, this position can adversely affect pulmonary mechanics, leading to decreased lung compliance and increased airway pressure. Changes in lung ventilation and perfusion distribution are well-documented with positional alterations, particularly in the lateral and prone positions. When the patient turns to the prone position, lung compliance decreases and airway pressure increases (1). This change can be attributed to the gravitational redistribution of lung tissue, which leads to increased compression of the anterior lung regions. Since lung compliance is linearly related to the volume of aerated lung tissue (2), a reduction in lung compliance may reflect a decrease in ventilated tissue, potentially reducing ventilation efficiency (3).

Lung protective ventilation strategies (LPVS) play an important role in optimizing lung ventilation, preventing atelectasis, improving gas distribution and exchange, and minimizing lung injury (4–7). It includes low tidal volume (V_T_), alveolar recruitment maneuvers (ARMs), and the use of PEEP (8, 9). In recent years, the strategy of driving pressure-guided ventilation has been widely concerned. A recent study has highlighted the importance of driving pressure in mechanical ventilation as it is a key indicator of lung stress and potential postoperative pulmonary complications (PPCs) (10).

Prolonging the end-inspiratory pause (EIP), while maintaining an adequate expiratory time, can increase the time available for alveolar gas exchange and reduce the dead space, which has obvious benefits for optimizing alveolar effective ventilation and enhancing gas exchange in surgical or intensive care patients (8, 11, 12). The duration of EIP is generally tailored according to the patient’s condition and is usually set between 10 and 40% of the total inspiratory time in recent published research studies (8, 13–15). A recent study suggested that a tailored open lung approach combined with a longer EIP (30% of the total inspiratory time) was associated with higher respiratory system compliance (C_RS_) and lower driving pressure during non-laparoscopic major surgery (8).

At present, there are no published research studies that reported the effect of LPVS combined with individualized EIP guided by driving pressure on respiratory mechanics in patients undergoing prone spinal surgery. In this study, we examined the effect of different EIP (10% of the total inspiratory time or individualized EIP) combined with LPVS on pulmonary compliance in patients undergoing prone spinal surgery to determine whether individualized EIP was effective in improving lung ventilation and optimizing oxygenation.

## Methods

### Participants

This prospective, randomized controlled trial was approved by the hospital Ethics Committee (2023–235) and registered with the Chinese Clinical Trial Registry (No. ChiCTR2300074398). All participants provided written informed consents prior to inclusion.

Patients who were scheduled for prone spinal surgery under general anesthesia in our hospital form August to October 2023 were recruited. Inclusion criteria were age 18 years or older, American Society of Anesthesiologists (ASA) physical status of I to III, and New York Heart Association classification of I to II. Exclusion criteria were any lung disease (acute lung injury, chronic obstructive pulmonary disease, asthma, and airway stenosis), Body Mass Index (BMI) below 18 or above 28, heavy smokers, a history of thoracic surgery, or psychiatric disorder.

A pilot study with 10 patients was performed to measure the difference in C_RS_ between the two groups to estimate the required sample size. We determined that the mean difference between the groups was 6 mL·cmH_2_O^−1^. Using PASS 15.0 software and assuming a standard deviation of 6, we calculated that 17 participants in each group were necessary to demonstrate a statistically significant difference with a power of 0.80 and *α* of 0.05. Taking into account an expected dropout of 10%, we needed a total of 38 patients.

### Randomization and blinding technique

Participants eligible for the study were randomly assigned to either the control group or the study group according to a randomization list generated by a computer random-number generator on the day before surgery. The intervention details were kept in a non-transparent, closed and numbered envelop. An investigator who administered the intervention as specified in the envelope and recorded mechanical ventilation parameters was not involved in designing the protocol and not blinded to group allocation. Data collectors, outcome assessors, an independent statistician, other clinical staff (including anesthesiologists and surgeons not involved in the intervention), and patients were kept unaware of the group allocation to ensure the integrity of the blinding process.

### Anesthesia management and standard procedure

No premedication was administered to any of the patients. In the operating room, standard monitoring including heart rate (HR), blood pressure (BP), temperature (T), pulse oxygen saturation (SpO_2_), and electrocardiogram (ECG) was recorded throughout the surgery. Invasive arterial catheterization was performed under local anesthesia, and biochemistry analyses (time basal) were collected on 21% inspired fraction of oxygen (F_i_O_2_). All patients were pre-oxygenated via a facial mask for 3 min. Anesthesia induction involved the intravenous administration of midazolam (0.02–0.04 mg·Kg^−1^), propofol (1.5–2.0 mg·Kg^−1^), sufentanil (0.3–0.5 μg·Kg^−1^), and rocuronium (0.6–0.8 mg·Kg^−1^). Mechanical ventilation was performed after endotracheal intubation. The ventilatory settings were used as follows: volume-controlled ventilation (VCV) settings, inspiration: expiration (I: E) ratio of 1:2, tidal volume (V_T_) of 7 mL·Kg^−1^ of predicted body weight (PBW), respiratory rate (RR) of 13 breaths per minute, EIP of 10% of the total inspiratory time (anesthesia machine default settings), PEEP at 4cmH_2_O, and maintained end-tidal CO_2_ pressure (P_ET_CO_2_) at 35–45 mmHg. All ventilatory settings remained unchanged, except for EIP. We recorded the basal mechanical ventilation parameters (Time a) before changing the position. Anesthesia was maintained with propofol 2–4 mg·Kg^−1^, remifentanil 0.05–0.25 μg·Kg^−1^·min^−1^, and 1–1.5% sevoflurane to maintain bispectral index (BIS) value between 40 and 60 throughout the surgery. Sufentanil and rocuronium were administered intermittently when required. After changing the position, we confirmed the correct position of the endotracheal tube by auscultating both lungs immediately.

### Ventilation protocol

**Figure 1:**
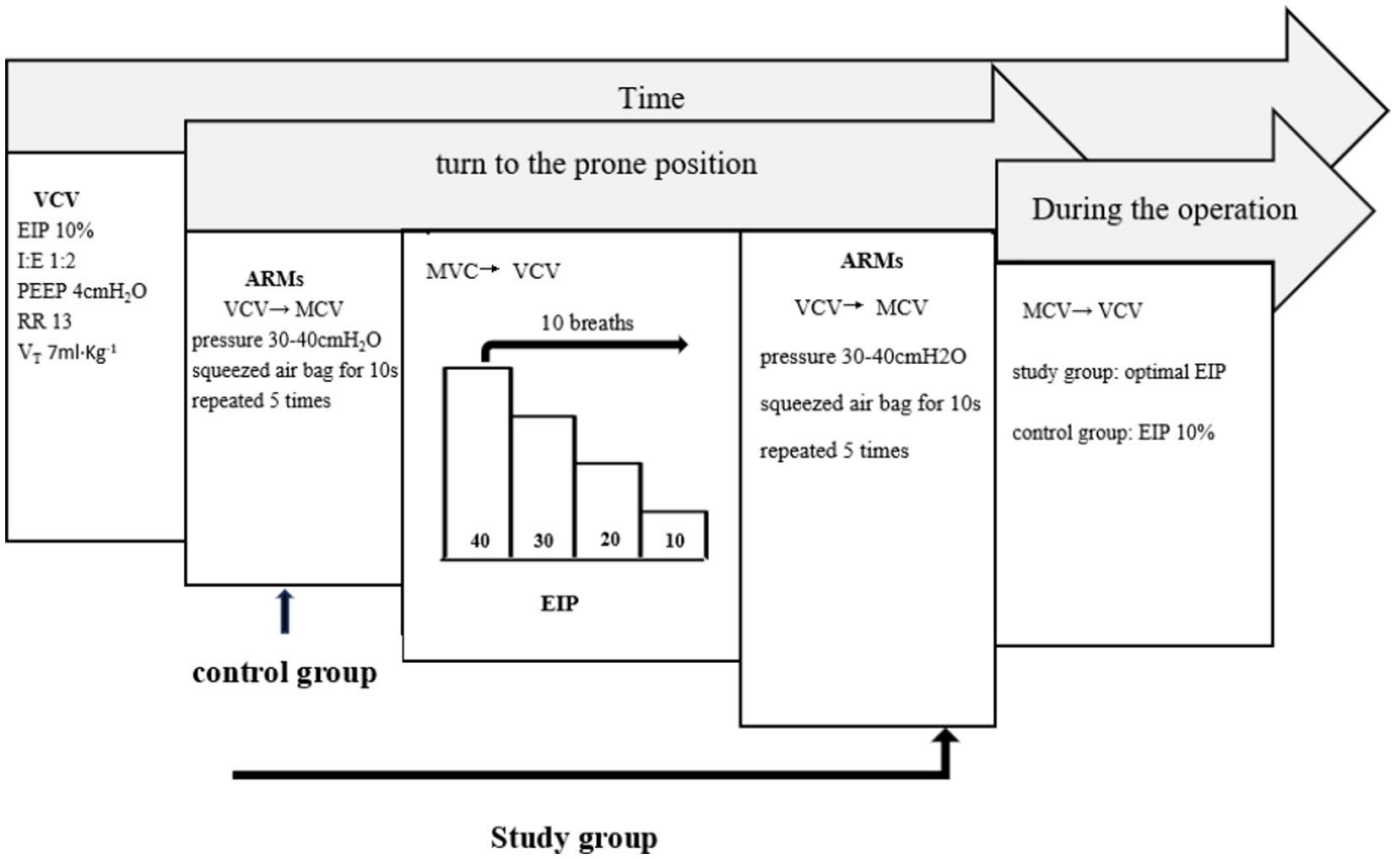
Ventilation protocol. VCV, volume-controlled ventilation; EIP, end- inspiratory pause; I: E, inspiration: expiration; PEEP, positive end expiratory pressure; RR, respiratory rate; Vt, tidal volume; ARMs, alveolar recruitment maneuvers; MCV, manually controlled ventilation.

The arterial blood gas sample and mechanical ventilation metrics were collected 2 min after the patient turned to the prone position but before ARMs (Time 0). The ventilation protocol was performed as follows (Figure 1): An ARMs were performed by changing VCV to manually controlled ventilation and adjusting the pressure to 30–40 cmH_2_O. Airway pressure was sustained by squeezing the air bag for 10 seconds, and this procedure was repeated five times. If any hemodynamic instability (mean arterial pressure changes more than 20% of the baseline value) appeared during the period of the ARMs, the ARMs were interrupted and vasoactive agents are administered if necessary. After hemodynamic stabilization, a new ARM was resumed. After ARMs were accomplished, the ventilator mode was reset to VCV. In the study group, the EIP was set at 40% of the total inspiratory time and then decreased in steps of 10% every 10 breaths until the EIP reached 10%. The lowest EIP corresponding to the minimal driving pressure (the difference between the plateau pressure and the level of PEEP) was considered to be the optimal EIP. Once the optimal driving pressure is determined, a new ARM was performed immediately. Conversely, the EIP remained 10% of the total inspiratory time (default setting of anesthesia machine) in the control group. Arterial blood gas and mechanical ventilation data were recorded 2 min after ARMs in the control group or after titration of EIP in the study group (Time 1) and 1 h after changing the position (Time 2). Tracheal suction and ARMs were performed every half hour during operation. All patients were transferred to the post-anesthesia care unit (PACU) immediately after operation.

### Data collection and outcomes

**Figure 2:**
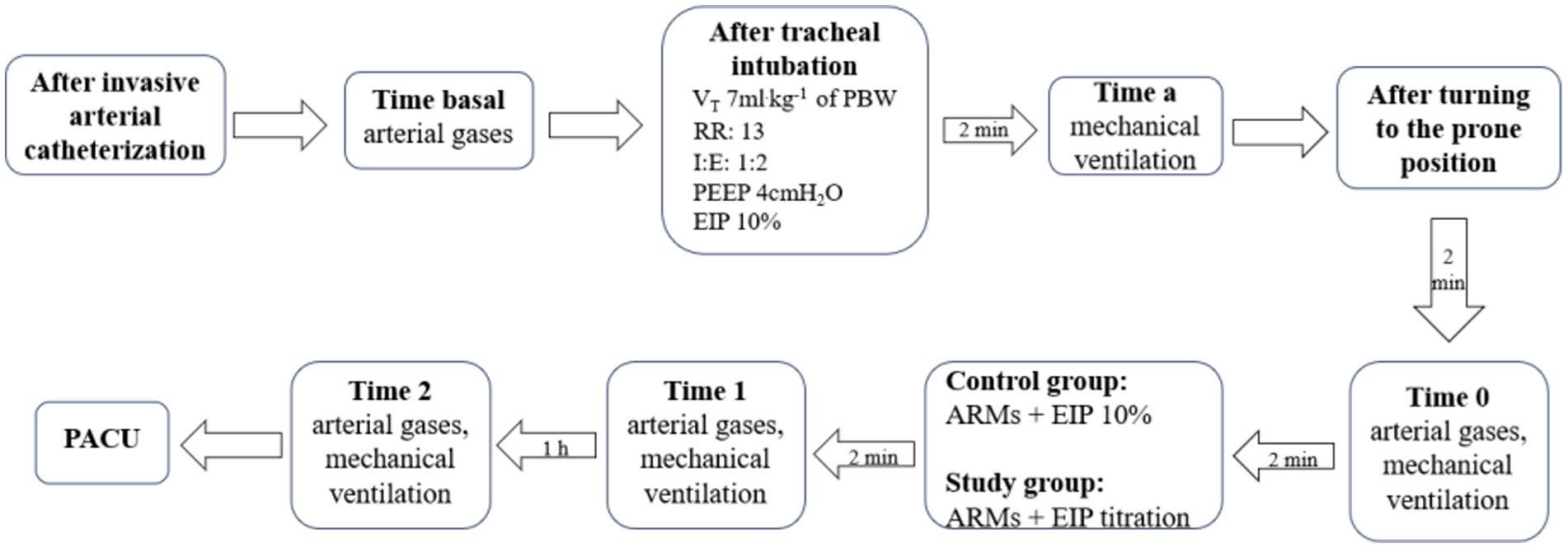
Study protocol and data collection times. Vt, tidal volume; PBW, predicted body weight; RR, respiratory rate; I: E, inspiration: expiration; PEEP, positive end expiratory pressure; EIP, end- inspiratory pause; ARMs, alveolar recruitment maneuvers; PACU, post-anesthesia care unit.

Apart from baseline characteristics such as age, gender, BMI, ASA physical status, duration of surgery, and anesthesia for both groups, all study variables were recorded at five different time points (Figure 2). This study compared the effect of a fixed EIP with an individualized EIP guided by driving pressure on respiratory parameters and arterial blood gas during the prone spinal surgery. We also recorded the occurrence of PPCs within 3 days of surgery, identified by three or more new symptoms, including cough, increased sputum, dyspnea, chest pain, a temperature exceeding 38°C, and heart rate over 100 beats per minute (15).

The primary outcome of the study was C_RS_ (calculated as V_T_ divided by driving pressure) in both groups at Time 1. The secondary outcome measurements were C_RS_ at other time points except Time1, driving pressure, plateau pressure, and blood gas analysis including PaO_2_, PaCO_2,_ and oxygenation index (P_aO2_/F_iO2_) in both groups.

### Statistical analysis

All statistical analyses were performed with IBM SPSS Statistics 26.0, and *a p***-**value of less than 0.05 (two-tailed) was considered statistically significant. We used mean (standard deviation, SD) or median (interquartile range, IQR) for quantitative variables. Then, *t-*test or Wilcoxon test was used for comparison between both groups accordingly. Paired Wilcoxon test for paired samples was used to compare intragroup quantitative variables at different positions (intragroup comparisons). Qualitative variables were represented by number (proportion) and Fisher’s exact test or Pearson’s chi-squared test was used for comparison between two groups.

## Results

**Figure 3:**
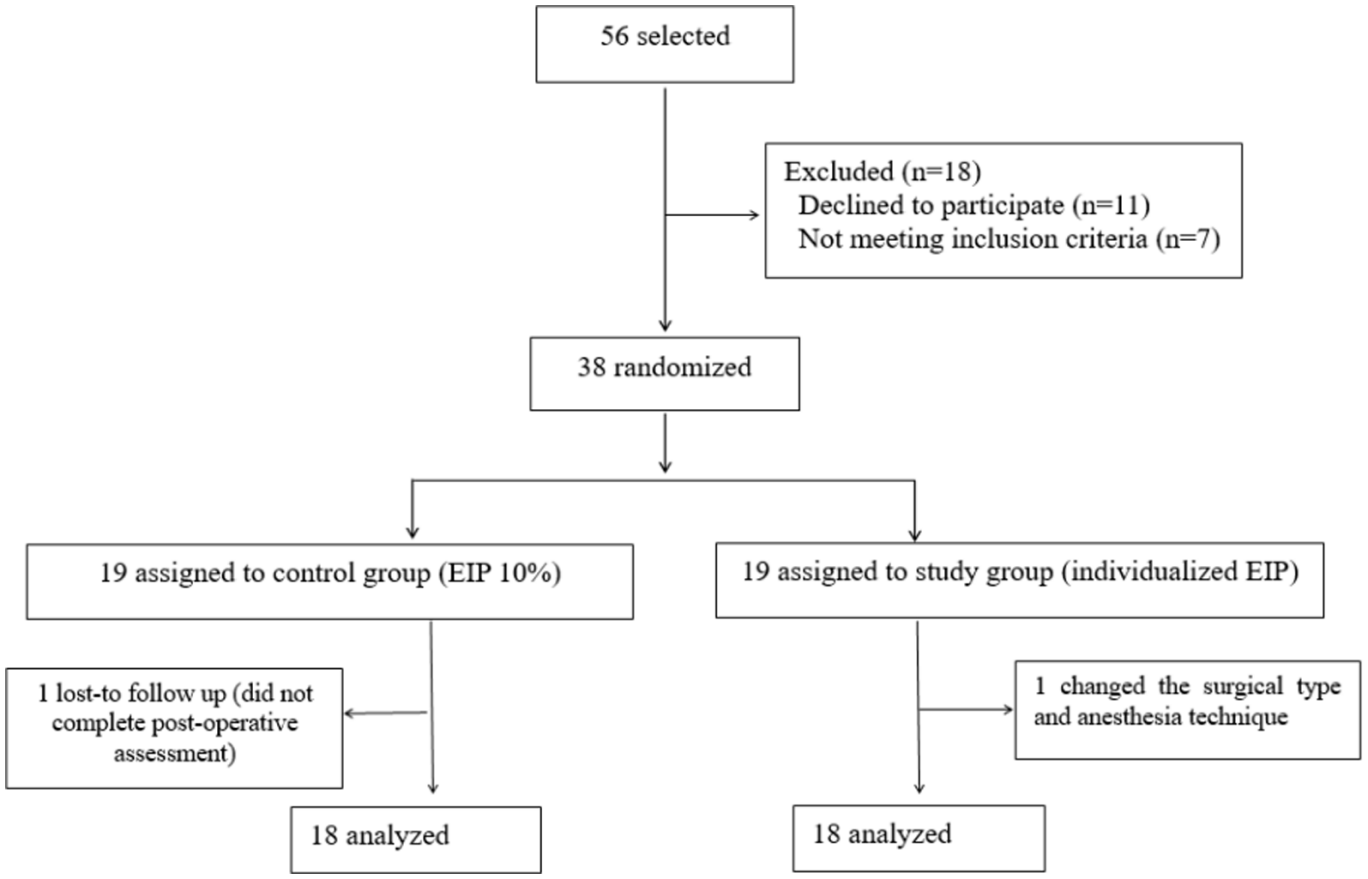
Flowchart of participants throughout the study. EIP, end- inspiratory pause.

A total of 56 patients scheduled for elective prone spinal surgery were initially assessed for eligibility. A total of 11 patients declined to participate, and 7 patients were excluded because of exclusion criteria. Finally, 38 patients were randomized into two groups. The registration flow chart is shown in Figure 3, and the demographic characteristics including surgical and anesthetic characteristics of both groups are shown in Table 1. The ventilatory parameters and arterial blood gases data of the two groups after changing the position (Time 0) are detailed in Table 2.

**Table 1:** Demographic data and basal ventilatory parameters and arterial blood gases measurements in both groups.

Characteristics	Group C (*n* = 18)	Group E (*n* = 18)	*P*
Age ( $\bar{x\;}\pm s$ , years)	56.6 ± 7.7	54.2 ± 6.9	0.324
Gender (female/male, n)	10/8	8/10	0.740
BMI ( $\bar{x\;}\pm s$ , kg/m^2^)	24.3 ± 2.0	23.2 ± 1.6	0.061
ASA grade (I/II/III, n)	5/11/2	7/9/2	0.887
NYHA grade (I/II, n)	5/13	7/11	0.725
PaO_2_ basal (mmHg)	82.1 ± 8.5	84.7 ± 10.0	0.407
PaCO_2_ basal (mmHg)	38.0 ± 2.7	39.3 ± 3.8	0.256
P_aO2_/F_iO2_ basal	390.6 ± 40.7	403.1 ± 47.8	0.407
P_plat_ basal	13(12,14)	12(12,13)	0.462
P_driv_ basal	9(8,10)	8(8,9)	0.462
P_peak_ basal	16(15,17)	15(15,16)	0.226
C_RS_ basal	48.8 ± 7.2	52.3 ± 8.1	0.176
Operation time ( $\bar{x\;}\pm s$ , min)	160.4 ± 31.2	166.8 ± 25.7	0.508
Anesthesia time ( $\bar{x\;}\pm s$ , min)	187.2 ± 34.9	193.3 ± 30.9	0.578

BMI, Body Mass Index; ASA, American Society of Anesthesiologists; NYHA, New York Heart Association; P_plat_, plateau pressure; P_driv_, driving pressure; P_peak_, peak pressure; C_RS_, compliance of the respiratory system.

**Table 2:** Ventilatory parameters and arterial blood gases after turning to the prone position in both groups (Time 0).

Characteristics	Group C (*n* = 18)	Group E (*n* = 18)	*P*
PaO_2_ (mmHg)	228(217, 249)	237(227, 252)	0.542
PaCO_2_ (mmHg)	40.5(37, 43)	39(37, 43)	0.938
P_aO2_/F_iO2_	459(434, 488)	469(458, 496)	0.606
P_plat_	15(13, 15)	14(13, 14)	0.279
P_driv_	10.5(9, 11)	9.5(9, 10)	0.279
P_peak_	18(17, 18)	17(16, 18)	0.323
C_RS_	41.8 ± 5.7	44.6 ± 5.6	0.141

P_plat_, plateau pressure; P_driv_, driving pressure; P_peak_, peak pressure; C_RS_, compliance of the respiratory system.

### Primary outcome

Compared with fixed EIP, the C_RS_ was significantly higher (*P* < 0.05) in the study group (Table 3). In addition, the optimal EIP for patients in the study group was determined to be 30% of the total inspiratory time.

**Table 3:** Ventilatory parameters and arterial blood gases after applying a recruitment maneuver and an individualized EIP (Time 1).

Characteristics	Control group (*n* = 18)	Study group (*n* = 18)	*P*
EIP (%)	10	30	
PaO_2_ (mmHg)	239(223, 260)	256(244, 263)	0.143
PaCO_2_ (mmHg)	38(36, 42)	36.5(35, 41)	0.355
P_aO2_/F_iO2_	489.5(451, 522)^*^	526.3(487.8, 543.8)^*^	0.029
P_plat_	14(13, 15)^*^	12(12, 13) ^*^	0.000
P_driv_	10(9, 11) ^*^	8(8, 9) ^*^	0.000
P_peak_	17(16, 18)	18(17, 18)	0.192
C_RS_	43.7 ± 5.5 ^*^	53.4 ± 6.1 ^*^	0.000

EIP, end- inspiratory pause; P_plat_, plateau pressure; P_driv_, driving pressure; P_peak_, peak pressure; C_RS_, compliance of the respiratory system. *Compared with the control group, the difference was significant at 0.05 level.

### Secondary outcomes

**Figure 4:**
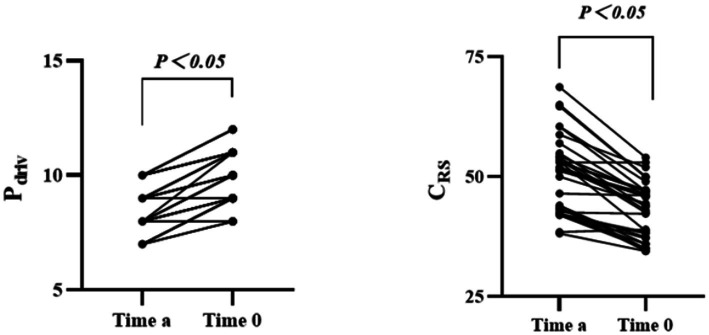
Comparison of ventilatory parameters before and after changing position (*n* = 36). Pdriv, driving pressure; CRS, compliance of the respiratory system.

Through paired Wilcoxon test, we found that the prone position was associated with a decrease in C_RS_ and an increase in driving pressure (*P* < 0.05) (Figure 4).

**Figure 5:**
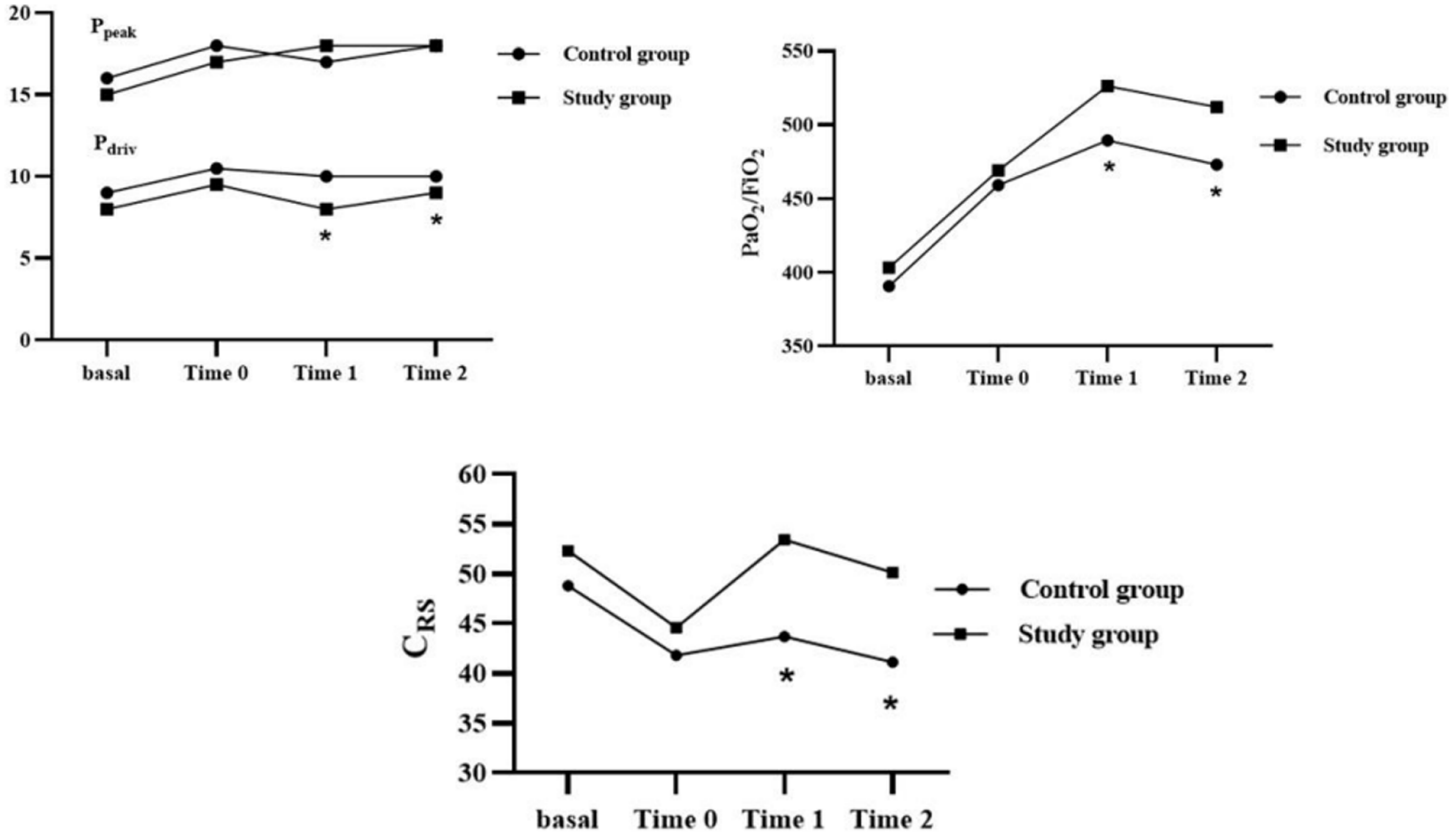
Intraoperative ventilatory parameters and arterial blood gases variations over time in two groups. Pdriv, driving pressure; Ppeak, peak pressure; CRS, compliance of the respiratory system; *: compared with the control group, the difference was significant at 0.05 level.

In the study group, we also observed a significant increase in oxygenation (*P* < 0.05) and a significant decrease in driving pressure and plateau pressure (*P* < 0.05) (Table 3). However, no significant differences were observed in PaO_2_, PaCO_2,_ and peak airway pressure (*P* > 0.05). As illustrated in Figure 5, patients in the study group still maintained higher C_RS_ and better oxygenation, as well as lower driving pressure throughout the surgery (*P* < 0.05). We also found that, even if ARMs helped improve C_RS_, LPVS combined with individualized EIP resulted in better lung condition and lung efficiency (Figure 5).

No PPCs were observed in all patients within 3 days after surgery.

## Discussion

In this study, we investigated the effect of individualized EIP guided by driving pressure on respiratory mechanics in patients during prone spinal surgery. We found that while LPVS could improve lung condition, using LPVS combined with individualized EIP guided by driving pressure could provide additional advantages for respiratory system mechanics, such as higher lung compliance, lower driving pressure, and better oxygenation. The benefits did not immediately disappear during surgery.

It is well known that changes in body position, particularly when changing to the lateral or prone position, lead to variations in lung ventilation and blood flow. Our study indicates that prone position is related to significantly reducing CRS and increasing driving pressure. This observation aligns with a recent study, which reported that the prone position was associated with a decrease in C_RS_ (1). Moreover, higher intraoperative driving pressure has been shown to be independently associated with the major postoperative pulmonary complications (16, 17). These complications include pulmonary edema, lung injury, barotrauma, and pneumonia.

Compared with fixed EIP (10% of the total inspiratory time), individualized EIP significantly decreased driving pressure and improved C_RS_ in the prone position. Meanwhile, we observed significant improvements in C_RS_ and driving pressure during surgery in patients in the study group. These benefits attributable to an individualized and longer EIP would depend on the potential reduction of overdistended alveoli (8). In our study, the optimal EIP for all subjects in the study group was determined to be 30% of the total inspiratory time. A previous study also found that an open lung approach strategy (including a low V_T_, ARMs, and the use of individualized PEEP) combined with a longer EIP (30% of the total inspiratory time) was associated with a higher C_RS_ and a lower driving pressure (8). This effect is due to redistribution of resident gas to alveoli with longer time constants during the static phase of inspiration, thus reducing the overdistended alveolar, which suggests higher C_RS_ and lower driving pressure (8). Recently, driving pressure has emerged as a critical indicator in the field of lung protection. The driving pressure is the force responsible for inflating the alveoli (18, 19). As mentioned above, driving pressure is independently related to postoperative pulmonary complications, not only in intensive care patients (19, 20) but also in the surgical patients (16, 17).

In this study, we also found that individualized EIP was superior to fixed EIP for improving oxygenation in the prone position. We speculate that this improvement in oxygenation could be attributed to the prolonged EIP extending the ventilation time. The mechanism is supported by the Uttman and Jonson’s model, which suggests that a longer EIP may related to an increase in mean distribution time (MDT), allowing the inspired gas to remain longer in the zone of gas exchange (21). According to Uttman and Jonson, a greater MDT may allow a reduction of airway dead space, although they noted that the benefits diminish as MDT increases (21). We can assume that the more effective alveolar ventilation is, the smaller margin of improvement is. Indeed, a previous study has confirmed that changing EIP from 10 to 30% reduces airway dead space (22). However, another recent study reported that oxygenation did not increase significantly with increasing EIP, and the authors attributed this difference to the use of an open lung strategy (ARMs combined with individualized PEEP) which already optimized alveolar ventilation (8). In our study, we used lower level of PEEP instead of titrating PEEP which may explain the different outcomes observed.

### Limitations

This study has several limitations. First of all, we did not perform preoperative lung function tests, relying instead on patient’s medical history, clinical evaluations, and chest CT to rule out pulmonary disease. In fact, we exclude patients with lung disease, which made it impossible for us to extend our conclusions to patients with respiratory dysfunction. Another limitation of this study was that we did not assess the ventilation parameters and arterial blood gas data in patients in the PACU, and longer after surgery. In addition, because the individualized EIP of the study group was titrated according to the driving pressure, it would be difficult to blind the investigator who performed the ventilation protocol. Finally, to ensure the sterility of the surgical field, no intraoperative imaging tests were used to monitor lung ventilation, and these tests should be considered for future studies.

## Conclusion

In conclusion, the individualized end-inspiratory pause guided by driving pressure effectively optimizes respiratory mechanics during prone spinal surgery. This strategy effectively improves pulmonary compliance and optimizes oxygenation, thereby contributing to better perioperative respiratory outcomes.

## Data Availability

The raw data supporting the conclusions of this article will be made available by the authors, without undue reservation.
