# Correction: Effect of individualized end-inspiratory pause guided by driving pressure on respiratory mechanics during prone spinal surgery: a randomized controlled trial

**DOI:** 10.3389/fmed.2025.1631254

**Published:** 2025-06-13

**Authors:** Ting Zhang, Feng Lv, Shuangyu He, Yuntian Zhang, Li Ren, Juying Jin

**Affiliations:** Department of Anesthesiology, The First Affiliated Hospital of Chongqing Medical University, Chongqing, China

**Keywords:** end-inspiratory pause, driving pressure, lung protective ventilation strategy, pulmonary compliance, prone spinal surgery

In the published article, there was an error regarding the affiliation(s) for all authors. The affiliation was incorrectly listed as:

“Department of Anesthesiology, First Affiliated Hospital of Chongqing Medical University, Chongqing, China”.

The correct affiliation should be:

“Department of Anesthesiology, The First Affiliated Hospital of Chongqing Medical University, Chongqing, China”.

The authors apologize for this error and state that this does not change the scientific conclusions of the article in any way. The original article has been updated.

